# NOD-like receptor X1 functions as a tumor suppressor by inhibiting epithelial-mesenchymal transition and inducing aging in hepatocellular carcinoma cells

**DOI:** 10.1186/s13045-018-0573-9

**Published:** 2018-02-26

**Authors:** Bo Hu, Guang-Yu Ding, Pei-Yao Fu, Xiao-Dong Zhu, Yuan Ji, Guo-Ming Shi, Ying-Hao Shen, Jia-Bin Cai, Zhen Yang, Jian Zhou, Jia Fan, Hui-Chuan Sun, Ming Kuang, Cheng Huang

**Affiliations:** 1 Department of Liver Surgery and Transplant, Liver Cancer Institute and Zhongshan Hospital, Fudan University, 136 Yi Xue Yuan Rd, Shanghai, 200032 China; 2 Key Laboratory for Carcinogenesis and Cancer Invasion, Chinese Ministry of Education, Shanghai, China; 3 Department of Pathology, Zhongshan Hospital, Fudan University, Shanghai, China; 4 Department of Liver Surgery, The First Affiliated Hospital, Sun Yat-sen University, 58 Zhong Shan Rd 2, Guangzhou, 510080 China; 5 Key Laboratory of Computational Biology, CAS-MPG Partner Institute for Computational Biology, 320 Yue Yang Road, Shanghai, 200031 China

**Keywords:** Hepatocellular carcinoma, NLRX1, Epithelial-mesenchymal-transition, Tumor suppressor, Senescence, Transition, Tumor suppressor, Senescence

## Abstract

**Background:**

This study was performed to investigate the role of nucleotide-binding oligomerization domain (NOD)-like receptor X1 (NLRX1) in regulating hepatocellular carcinoma (HCC) progression.

**Methods:**

Expression levels of NLRX1 in clinical specimens and cell lines were determined by reverse transcription-polymerase chain reaction (RT-PCR) and western blot (WB). Transwell assays were conducted to evaluate the effect of NLRX1 on cell invasion, and flow cytometry was used to assess apoptosis. Expression patterns of key molecules in the phosphoinositide 3-kinase (PI3K)-AKT pathways were determined via WB. The effect of NLRX1 on cell senescence was evaluated with β-galactosidase assays. Kaplan-Meier analyses and Cox regression models were used for prognostic evaluation.

**Results:**

NLRX1 was downregulated in tumor tissue compared with adjacent normal liver tissue. Low tumor NLRX1 expression was identified as an independent indicator for HCC prognosis (recurrence: hazard ratio [HR] 1.87, 95% confidence interval [CI] 1.26–2.76, overall survival [OS] 2.26, 95% CI 1.44–3.56). NLRX1 over-expression (OE) significantly inhibited invasiveness ability and induced apoptosis in HCC cells. In vivo experiments showed that NLRX1 knock-down (KD) significantly promoted HCC growth. Mechanistically, NLRX1 exhibited a suppressor function by decreasing phosphorylation of AKT and thus downregulating Snail1 expression, which inhibited epithelial-mesenchymal-transition (EMT) in HCC cells. Moreover, NLRX1 OE could induce cell senescence via an AKT-P21-dependent manner.

**Conclusions:**

NLRX1 acted as a tumor suppressor in HCC by inducing apoptosis, promoting senescence, and decreasing invasiveness by repressing PI3K-AKT signaling pathway. Future investigations will focus on restoring expression of NLRX1 to provide new insights into HCC treatment.

**Electronic supplementary material:**

The online version of this article (10.1186/s13045-018-0573-9) contains supplementary material, which is available to authorized users.

## Background

Hepatocellular carcinoma (HCC) is the fifth most prevalent malignant disease and the third leading cause of cancer-related death worldwide [1]. Despite considerable improvements in systemic treatment for HCC in recent years, clinical outcomes remain unsatisfactory [2–4]. Approximately 19–25% of patients with HCC who undergo curative resection suffer recurrence within 1 year after treatment, and recurrence rate exceeds 70% at 5 years [5–8]. Identifying molecules involved in HCC progression may lead to novel treatments that improve patient outcomes.

The nucleotide-binding oligomerization domain (NOD)-like receptor (NLR) family comprises evolutionarily conserved components of the immune system that serve vital roles in immune defense and inflammation [9–11]. Most studies of NLR have focused on their pro-inflammatory functions, which result in caspase-1 activation and subsequent production of interleukin (IL) 1β (IL-1β) and IL-18 [12, 13]. However, recent investigations revealed that several NLR family proteins regulate cell proliferation, invasion, and survival [14, 15]. Moreover, some NLRs are reportedly involved in the mitogen-activated protein kinase and nuclear factor κB (NF-κB) signaling pathways [9, 16, 17], which were previously shown to induce several types of tumorigenesis. Therefore, it is important to explore whether NLRs contribute to HCC progression and assess their translational relevance in clinical practice.

NOD-like receptor X1 (NLRX1) is unique within the NLR family because it has inflammasome function and negatively regulates the expression of pro-inflammatory cytokines including IL-6 [18, 19]. Importantly, recent studies revealed NLRX1 as a critical regulator in tumorigenesis that serves as a suppressor in solid tumors including colorectal cancer via inhibiting of NF-κB signaling, type-I interferon production, reactive oxygen species production, and autophagy promotion [17, 20–22]. However, if and how it serves as a suppressor in HCC remain unclear.

The present study was conducted to explore the influence of NLRX1 on the biological function of HCC cells. We performed in vitro and in vivo experiments, and the prognostic significance of NLRX1 was assessed in clinical samples. The effects of NLRX1 in epithelial-mesenchymal transition (EMT), which contributes to cell invasion, as well as cell aging, which impacts apoptosis, were investigated. Proteomics approaches were used to identify the critical fragment of NLRX1 involved in regulating EMT and aging.

## Methods

### Patient specimens

From January to December 2008, 635 patients with HCC were recruited. Enrollment criteria were as follows: (a) definitive HCC diagnosis, (b) no prior cancer treatment, (c) complete resection of all tumor nodules with margins confirmed free of cancer by histologic examination, and (d) availability of complete clinicopathologic and follow-up data [5]. HCC diagnosis was based on histopathology. The Barcelona Clinic Liver Cancer (BCLC) staging system was used to assess tumor stage [2]. Tumor differentiation was determined according to the Edmondson grading system. Liver function was assessed with the Child–Pugh scoring system [2]. Approval for use of human subjects was obtained from the research ethics committee of Zhongshan Hospital. Informed consent was obtained from each subject.

### Follow-up

Patients were prospectively monitored by serum α-fetoprotein (AFP) testing, abdomen ultrasonography, and chest X-ray every 1–6 months as previously described [5]. Follow-up ended in August 2017. Time to recurrence (TTR) was defined as the interval between surgery and the diagnosis of any type of recurrence including intra- or extrahepatic recurrence as identified by magnetic resonance imaging or computed tomography. OS was defined as the interval between treatment and death of any cause or the last observation date.

### Cell lines and cell culture

Huh7, HepG2, and 7721 cell lines were purchased from the Cell Bank at the Institute of Biochemistry and Cell Biology, China Academy of Science (Shanghai, China). MHCC97L, MHCC97H, and HCCLM3 cell lines were previously generated in our institute. All cell lines were cultured in Dulbecco’s modified Eagle’s medium (DMEM) containing 10% fetal bovine serum (FBS) supplemented with 100 IU/mL penicillin and 100 μg/mL streptomycin and incubated at 37 °C in a humidified atmosphere with 5% CO_2_. All cell culture reagents were obtained from Life Technologies (Thermo Fisher Scientific, Waltham, MA, USA).

### Apoptosis evaluation

Cell apoptosis was analyzed by flow cytometry using annexin V-fluorescein isothiocyanate (FITC). Apoptosis detection kits (BD Biosciences, San Jose, CA, USA) were used according to the manufacturer’s protocol. Briefly, cells exposed to different treatments were harvested and suspended in binding buffer. An aliquot of 100 μL was incubated with 5 μL annexin V-FITC and 5 μL propidium iodide for 15 min in the dark, and 400 μL binding buffer (1×) was added to each sample. The stained cells were analyzed by flow cytometry within 1 h.

### Immunofluorescence

HCCLM3 cells were fixed in 4% paraformaldehyde and blocked with 5% bovine serum albumin. Afterwards, 0.1% Triton was used for permeabilization followed by blocking with 5% bovine serum albumin. Then, FITC-conjugated mouse anti-human NLRX1 antibodies (1:30; BioLegend, San Diego, CA, USA) were added and incubated overnight at 4 °C. HCCLM3 cells were also counterstained with DAPI (Sigma-Aldrich, St. Louis, MO, USA). Images were captured using an IX-71 fluorescent microscope (Olympus, Tokyo, Japan).

### RNA isolation and analysis

Total RNA extraction was conducted by RNeasy mini kit (Qiagen, Hilden, Germany), and cDNA was synthesized via Quantitect Reverse Transcription Kit (Qiagen) according to the manufacturer’s instructions. Target genes were quantified using FastStart Universal SYBR Green Master (Roche Diagnostics, Basel, Switzerland), and DNA amplification was carried out using a LightCycler 480 (Roche Diagnostics). The relative quantities of target gene mRNAs compared to an internal control were determined using the ΔCq method. Reverse transcription-polymerase chain reaction (RT-PCR) conditions were as follows: 5 min at 95 °C, followed by 40 cycles of 95 °C for 10 s and 60 °C for 60 s. GAPDH was used as an internal control. Primers and probes are listed as follows: NLRX1, F: 5′-CGACCAGATGATCGTATCC-3′ R: 5′-TGCGTCACTGAGGTGTTTCCTGCC-3′; E-cadherin, F: 5′-TTGCTACTGGAACAGGGACAC-3′ R: 5′-CCCGTGTGTTAGTTCTGCTGT-3′; N-cadherin, F: 5′-TTATCCTTGTGCTGATGTTTGTG-3′ R: 5′-TCTTCTTCTCCTCCACCTTCTTC-3′; Vimentin, F: 5′-CCTTGACATTGAGATTGCCACCTA-3′ R: 5′- TCATCGTGATGCTGAGAAGTTTCG-3′; Snail1, F: 5′- TCCAGAGTTTACCTTCCAGCA -3′ R: 5’-CTTTCCCACTGTCCTACTCTG -3′; Twist1, F: 5’-GTCCGCAGTCTTACGAGGAG-3′ R:5′-GTCTGAATCTTGCTCAGCTTGTC-3′; beta-actin, F: 5′-CTGAGGACAAGCCACAAGATTA-3′ R: 5′-ATCCACCAGAGTGAAAAGAACG-3′.

### Western blot (WB) analysis

Protein from HCC cells were lysed in complete radioimmunoprecipitation assay buffer for WB analyses. All protein lysates were quantified using a quantitative bicinchoninic acid protein assay. A total of 30 μg protein was mixed in sodium dodecyl sulfate (SDS) loading dye containing 20 mg/mL dithiothreitol reducing agent, then boiled for 5 min. Proteins were separated by SDS-polyacrylamide gel electrophoresis using 4–12% Bis-Tris gels and wet transferred to nitrocellulose membrane (Bio-Rad, Hercules, CA, USA). Nitrocellulose membranes were blocked for 1 h with 10% non-fat milk and incubated overnight with primary antibodies, washed five times with Tris-buffered saline containing Tween, and incubated for 2 h at room temperature with the appropriate secondary antibodies. Then, protein expression was determined by chemiluminescent reagents (Thermo Fisher Scientific).

### Cell proliferation assays

For the proliferation assay, control or NLRX1-modulated HCC cells were aliquoted into a 96-well plate at 1000/100 μL per well. At the indicated time points, 20 μL of cell counting kit 8 (CCK-8) solution (Dojindo, Kamimashiki-gun, Kumamoto, Japan) was added to determine the number of viable cells in each well.

### Cell invasion and apoptosis assays

Cell invasion ability was evaluated by Transwell (Corning, Corning, NY, USA) assays as previously described. Briefly, HCC cells subjected to different treatments were collected and washed with phosphate-buffered saline (PBS). Next, 10^5^ cells were seeded in the upper chamber with a MatriGel-coated membrane (dilution 1:6), and the lower chambers were supplied with DMEM containing 10% FBS to act as chemo-attractant. After 24 or 48 h of incubation at 37 °C, cells that had already migrated or invaded to the lower surface of membrane were fixed with 4% methanol, stained with crystal violet, and counted in 10 random × 200 microscopic fields per sample.

Cell apoptosis was analyzed by flow cytometry using annexin V-FITC. Apoptosis Detection Kits (BD Biosciences) were used according to the manufacturer’s protocol. Briefly, cells were harvested and suspended in binding buffer (1×). An aliquot of 100 μL was incubated with 5 μL annexin V-FITC and 5 μL propidium iodide for 15 min in the dark, and 400 μL binding buffer (1×) was added to each sample. The stained cells were analyzed by flow cytometry within 1 h.

### Tissue MicroArray (TMA) and immunohistochemistry

The resected specimens were embedded in paraffin and stored at 4 °C. The construction of the TMA and immunohistochemistry protocol were described previously [9]. Briefly, immunohistochemical staining was performed using the avidin-biotin-peroxidase complex method. After rehydration and microwave antigen retrieval, primary anti-human-NLRX1 antibodies were applied to slides for overnight incubation at 4 °C. Then, secondary antibody incubation was conducted at 37 °C for 30 min. Staining was performed with 3′3-diaminobenzidine tetra hydrochloride, and counterstaining was performed with Mayer’s hematoxylin. We included negative control slides with the primary antibodies omitted in all assays. Immunohistochemical staining was independently assessed by two pathologists.

### Cignal Finder RTK signaling 10-Pathway Reporter array

Cignal Finder RTK signaling 10-Pathway Reporter array was used to uncover the potential down-stream signaling pathway controlled by NLRX1. Control, NLRX1-KD Huh7 cells and control, NLRX1-OE HCCLM3 cells were subsequently transfected with a mixture of a transcription factor-responsive firefly luciferase reporter and a constitutively expressing Renilla construct. The relative activity of each pathway was decided by luciferase/Renilla and normalized by untreated controls. Experiments were performed in triplicates.

### In vivo animal assays

For the mouse xenograft model, 6-week-old male nude mice were purchased from the Chinese Science Academy (Shanghai, China). Mice were subcutaneously implanted with Huh7 cells infected with lentivirus (Huh7, Huh7-NLRX1^KD^; 3 × 10^6^). Tumor volume was measured twice a week, and tumor growth was calculated as the follow equation: larger diameter × (small diameter)^2^/2. Five weeks after HCC cell injection, the mice were sacrificed, and tumor tissues were resected for hematoxylin and eosin staining.

### Plasmid constructs, transfection, and retrovirus infection

The Flag-Nlrx1, Flag-Nlrx1R1, and Flag-Nlrx1R2 truncations were subcloned into pcDNA3.1 vectors. The shNlrx1 was subcloned into a PLKO.1 vector. To transiently express a given protein, cells were transfected with Lipofectamine (Life Technologies, Carlsbad, CA, USA). For 6-cm plates, a total of 6 μg DNA was used for transfection, whereas 12 μg DNA was used to obtain saturated effects. A retroviral vector pMSCV was used to stably knock-down (KD) NLRX1 or negative control (NC). The retrovirus was produced by 293 T packaging cells. HCC cells were infected with these viruses or control virus and then selected against puromycin for 3 days before use in assays.

### β-galactosidase activity

HCC cellular senescence was evaluated by detecting the activity of senescence-associated β-galactosidase (SA-β-gal) with Senescence β-Galactosidase Staining Kits (Beyotime, China) according to the manufacturer’s instruction. Briefly, cells were seeded into six-well plates, cultured with the kit reagents for 48 h, fixed for 15 min at room temperature with 1 mL fixative solution, and then washed three times with PBS. Next, the cells were incubated overnight at 37 °C with a staining solution mixture containing X-gal. After cells were rinsed with PBS, they were observed for the development of the blue coloration under a light microscope (× 400).

### QPCR array

Expression profiling was conducted using Affymetrix 3’ IVT Expression microarray. Briefly, total RNA was extracted using RNeasy Mini Kit (Qiagen), then first-strand cDNA was synthesized by reverse transcription. Afterwards, labeled aRNA was synthesized and purified for further hybridization. Raw data were obtained for further analysis.

### Statistical analysis

Statistical analyses were performed using SPSS 20.0 software (IBM, Armonk, NY, USA). Experimental values for continuous variables are expressed as the mean ± standard error of the mean. Chi-squared tests, Fisher’s exact probability tests, and Student’s *t* tests were used when appropriate to evaluate the significance of differences between groups. If variances within groups were not homogeneous, a nonparametric Mann–Whitney test or Wilcoxon signed-rank test was used. The relationships between NLRX1 expression and TTR or OS were analyzed using Kaplan–Meier survival curves and log-rank tests, respectively. *P* < 0.05 was considered statistically significant.

## Results

### NLRX1 is downregulated in tumor tissues and low expression indicates better prognosis in HCC

**Fig. 1:**
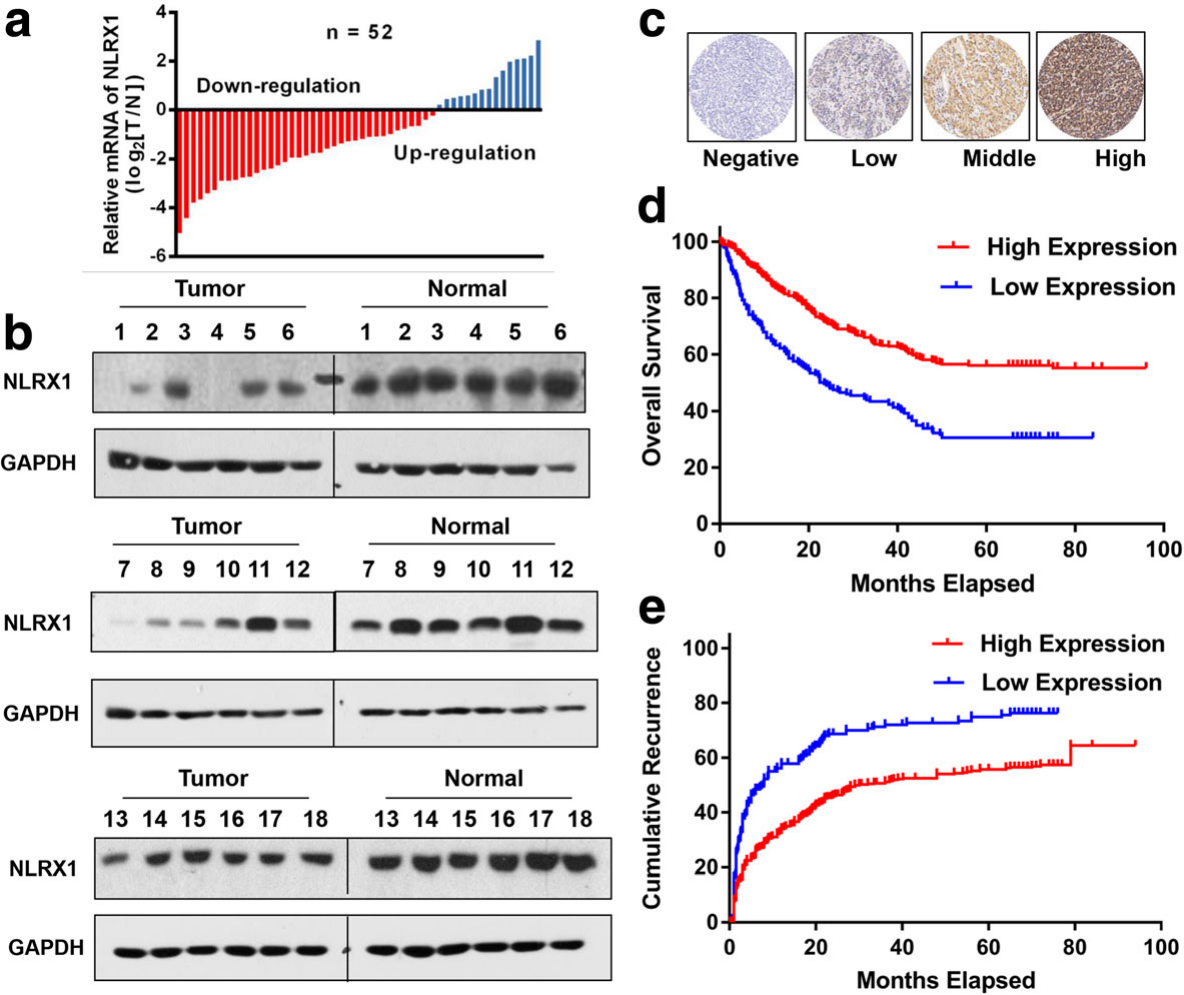
NLRX1 serves as a tumor suppressor in HCC. **a** RT-PCR results for paired tumor and adjacent normal liver tissues. **b** Western blot results for 18 paired tumor and adjacent normal liver tissues. **c** Typical IHC images. **d** Kaplan–Meier analysis for OS of patients with HCC according to NLRX1 expression status. **e** Kaplan–Meier analysis for RFS of patients with HCC according to NLRX1 expression status

First, we investigated the expression patterns of NLRX1 between HCC tumor tissues and paired adjacent normal liver tissues. RT-PCR showed that 44.23% (23/52) of tumors exhibited significantly lower expression of NLRX1 compared with paired normal liver tissues, while only 9.60% (5/52) of tumors showed NLRX1 upregulation (Fig. 1a). WBs confirmed downregulation of NLRX1 within tumor tissue (Fig. 1b).

To explore whether low NLRX1 expression was associated with disease prognosis, a TMA containing 575 patients who underwent curative resection was immunostained. NLRX1 expression status was stratified according to Fig. 1c and Table 1. For the 575 patients with HCC enrolled, the median TTR and OS were 20.00 months (range 0.60–59.00) and 45.00 months (range 2.50–59.00), respectively. Kaplan–Meier analysis revealed significantly shorter median OS in patients with low NLRX1 was compared with those whose status was high (12.80 months vs. not reached, *P* < 0.001; Fig. 1d). In addition, patients with low NLRX1 had significantly shorter TTR compared with patients with high expression (median 32.00 months vs. not reached, *P* < 0.001; Fig. 1e). Univariate analysis showed that TTR correlated with NLRX1 expression status, AFP level, tumor number, tumor size, satellite lesions, vascular invasion, differentiation, and BCLC stage (all *P* < 0.05, Table 2), while OS correlated with NLRX1 expression status, Child–Pugh score, AFP level, tumor size, satellite lesions, vascular invasion, differentiation, and BCLC stage (all *P* < 0.05, Table 2). Multivariate analysis revealed that NLRX1 expression status was an independent indicator for both TTR (hazard ratio [HR] 1.87, 95% confidence interval [CI] 1.26–2.76, *P* = 0.002; Table 3) and OS (HR 2.26, 95% CI 1.44–3.56, *P* = 0.001; Table 3). Collectively, these results indicate that NLRX1 might act as a tumor suppressor and be a useful prognostic biomarker in HCC.

**Table 1:** Correlation between clinicopathologic parameters and NLRX1 expression status

Clinical and pathologic indexes		High NLRX1		Low NLRX1		
		*n* = 382	%	*n* = 193	%	*P*
Age, years	≤ 50	149	36.3	70	33.7	0.586
	> 50	293	63.7	123	66.3	
Gender	Male	382	86.5	167	86.4	1.000
	Female	60	13.5	26	13.6	
HBsAg	Negative	87	28.0	54	19.7	0.023
	Positive	355	72.0	139	80.3	
With liver cirrhosis	No	7	1.60	3	1.60	1.000
	Yes	435	98.4	190	98.4	
GGT (U/L)	≤ 54	141	31.1	60	31.9	0.853
	> 54	301	68.9	133	68.1	
AFP (ng/mL)	≤400	280	50.8	98	63.3	0.004
	> 400	162	49.2	95	36.7	
Tumor number	Single	323	75.6	146	73.1	0.556
	Multiple	119	24.4	47	26.9	
Tumor size, cm	≤ 5	216	40.9	79	48.9	0.070
	> 5	226	59.1	114	51.1	
Tumor encapsulation	Complete	219	56.0	108	49.5	0.143
	None	223	44.0	85	50.5	
Edmondson stage	I-II	333	62.7	121	75.3	*0.002*
	II-IV	109	37.3	72	24.7	
BCLC stage	0-A	279	77.1	111	77.1	1.000
	B-C	83	22.9	33	22.9	
PVTT	None	278	48.2	93	62.9	*0.010*
	Yes	164	51.8	100	37.1	

*Abbreviations*: *AFP* α-fetoprotein, *BCLC* Barcelona Clinic Liver Cancer, *GGT* gamma-glutamyl transpeptidase, *HBsAg* hepatitis B virus surface antigen, *NLRX1* nucleotide-binding oligomerization domain-like receptor X1, *PVTT* portal vein tumor thrombus

**Table 2:** Univariate analyses of factors associated with survival and recurrence

Clinical and pathologic indexes		OS		RFS	
		HR (95%CI)	P	HR (95%CI)	*P*
Age, years	(≤ 50 vs. > 50)	0.98 (0.78,1.23)	0.873	0.82 (0.66,1.00)	0.055
Gender	(Male vs. female)	1.21 (0.87,1.69)	0.235	1.14 (0.85,1.53)	0.389
HBsAg	(Positive vs. negative)	2.95 (2.35,3.71)	*0.000*	2.83 (2.27,3.53)	*0.000*
Liver cirrhosis	(No vs. yes)	0.52 (0.17,1.63)	0.210	0.37 (0.119,1.154)	0.087
GGT (U/L)	(≤ 54 vs. > 54)	0.95 (0.76,1.20)	0.666	0.94 (0.76,1.16)	0.540
AFP (ng/mL)	(> 400 vs. ≤400)	1.23 (0.99,1.53)	0.064	1.33 (1.09,1.63)	*0.005*
Tumor number	(Multiple vs. single)	1.33 (1.05,1.69)	*0.017*	1.64 (1.32,2.04)	*0.000*
Tumor size, cm	(> 5 vs. ≤ 5)	1.09 (0.88,1.35)	0.436	1.11 (0.90,1.35)	0.328
Tumor encapsulation	(Complete vs. none)	0.58 (0.46,0.72)	*0.000*	0.67 (0.55,0.82)	*0.000*
Edmondson stage	(I-II vs. II-IV)	0.73 (0.58,0.92)	*0.008*	0.67 (0.54,0.82)	*0.000*
BCLC stage	(0-A vs. B-C)	0.57 (0.43,0.76)	*0.000*	0.49 (0.38,0.64)	*0.000*
PVTT	(None vs. yes)	0.47 (0.34,0.58)	*0.000*	0.56 (0.46,0.68)	*0.000*
NLRX1	(Positive vs. negative)	0.55 (0.44,0.67)	*0.000*	0.47 (0.38,0.59)	*0.000*

*Abbreviations*: *AFP* α-fetoprotein, *BCLC* Barcelona Clinic Liver Cancer, *CI* confidence interval, *HR* hazard ratio, *GGT* gamma-glutamyl transpeptidase, *HBsAg* hepatitis B virus surface antigen, *NLRX1* nucleotide-binding oligomerization domain-like receptor X1, *OS* overall survival, *PVTT* portal vein tumor thrombus, *RFS* recurrence-free survival

**Table 3:** Multivariate analyses of factors associated with survival and recurrence

Clinical and pathologic indexes		OS		RFS	
		HR (95% CI)	*P*	HR (95% CI)	*P*
HBsAg	(Positive vs. negative)	2.26 (1.16,4.41)	*0.017*	1.14 (0.53,2.43)	0.740
AFP (ng/mL)	(> 400 vs. ≤ 400)	–	–	1.12 (0.88,1.43)	0.360
Tumor number	(Multiple vs. single)	0.97 (0.49,1.91)	0.923	1.73 (1.02,2.95)	*0.043*
Tumor encapsulation	(Complete vs. none)	0.77 (0.59,0.99)	0.050	0.85 (0.67,1.09)	0.199
Edmondson stage	(I-II vs. II-IV)	–	–	0.73 (0.56,0.95)	*0.018*
BCLC stage	(0-A vs. B-C)	0.57 (0.28,1.14)	0.112	0.83 (0.48,1.46)	0.523
PVTT	(None vs. yes)	0.72 (0.55,0.94)	*0.018*	0.90 (0.70,1.17)	0.439
NLRX1	(Positive vs. negative)	0.51 (0.39,0.67)	*0.000*	0.58 (0.45,0.76)	*0.000*

*Abbreviations*: *AFP* α-fetoprotein, *BCLC* Barcelona Clinic Liver Cancer, *CI* confidence interval, *HR* hazard ratio, *GGT* gamma-glutamyl transpeptidase, *HBsAg* hepatitis B virus surface antigen, *NLRX1* nucleotide-binding oligomerization domain-like receptor X1, *OS* overall survival, *PVTT* portal vein tumor thrombus, *RFS* recurrence-free survival

### NLRX1 induces apoptosis and suppresses invasiveness in vitro

**Fig. 2:**
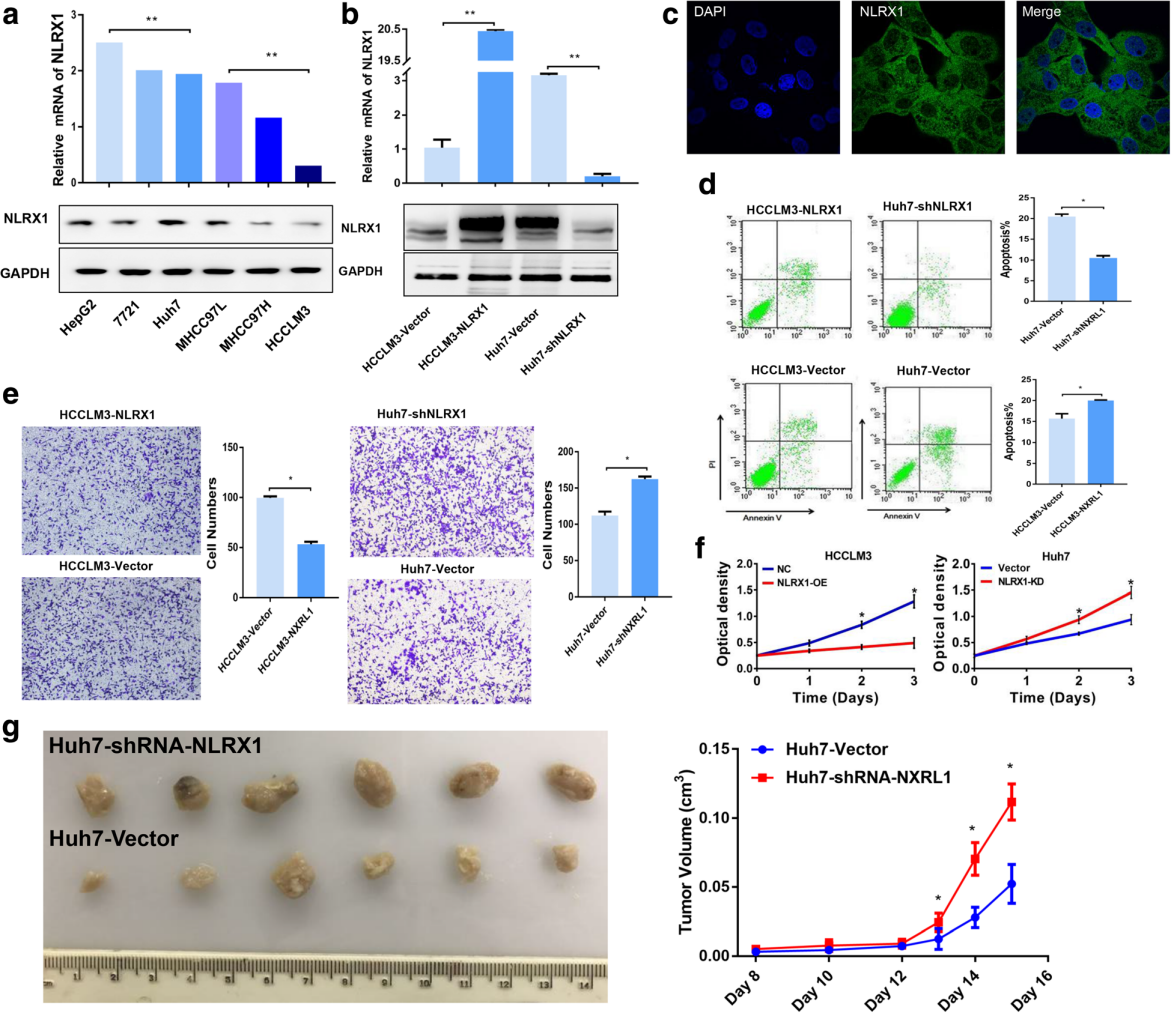
In vitro and in vivo functions of NLRX1 in HCC**. a** Expression of NLRX1 in 6 HCC cell lines determined by RT-PCR and WB. **b** Validation of NLRX1 modulation by RT-PCR and WB. **c** Immunofluorescent staining for NLRX1 in Huh7 cells. **d** Effect of NLRX1 on cell apoptosis as assessed by flow cytometry. **e** Evaluation of the effects of NLRX1 on HCC invasion by Transwell assays. **f** Evaluation of the effects of NLRX1 on tumor proliferation in vitro by CCK-8 assays. **g** Effect of NLRX1 on tumor proliferation in vivo. All in vitro experiments were conducted triplicate; one asterisk and three asterisks indicate *P* < 0.05 and *P* < 0.001, respectively

Next, we investigated the effect of NLRX1 on HCC cells. First, the expression status of NLRX1 in six HCC cell lines were evaluated with RT-PCR and WB. Interestingly, NLRX1 levels were low in cell lines with high metastasis potential, such as MHCC97H and HCCLM3, while it was high in cell lines with low metastasis potential, such as HepG2 and Huh7 (Fig. 2a). Based on these results, Huh7 and HCCLM3 cells were selected for further KD and over-expression (OE) experiments, respectively. The efficiencies of NLRX1 modification were validated by RT-PCR and WB (Fig. 2b). Intracellular localization of over-expressed NLRX1, which is mainly in the cytoplasm, was validated by immunofluorescent staining (Fig. 2c).

The effect of NLRX1 on tumor cell apoptosis was also investigated using flow cytometry. We found that NLRX1-OE significantly increased HCCLM3 cell apoptosis (*P* < 0.05). Conversely, NLRX1-KD significantly decreased the apoptosis rate of Huh7 cells (*P* < 0.05, Fig. 2d). Transwell assays were then conducted to explore the effect of NLRX1 on HCC cell invasiveness. The invasive potential of HCCLM3 cells was significantly decreased following NLRX1 OE (*P* < 0.05, Fig. 2e), while NLRX1-KD significantly promoted Huh7 cell invasive potential (*P* < 0.05, Fig. 2e).

### NLRX1 inhibits tumor growth in vitro and in vivo

To investigate the effect of NLRX1 on tumor growth, we first conducted CCK-8 assays. We found that NLRX1 overexpression could hinder cell proliferation in HCCLM3 cells, while knocking down NLRX1 in Huh7 resulted in significant higher proliferation rates (Fig. 2f). Next, 3 × 10^6^ Huh7-Vector (control) and KPNA3-KD Huh7 cells were subcutaneously implanted into nude mice. Mice injected with KPNA3-KD Huh7 developed significantly larger tumor volumes than those injected with Huh7-Vector cells after 5 weeks (Fig. 2g). Collectively, these findings indicate that NLRX1 effectively restrained HCC proliferation.

### NLRX1 suppresses EMT in HCC by inhibiting the phosphoinositide 3-kinase (PI3K)-AKT-Snail1 axis

**Fig. 3:**
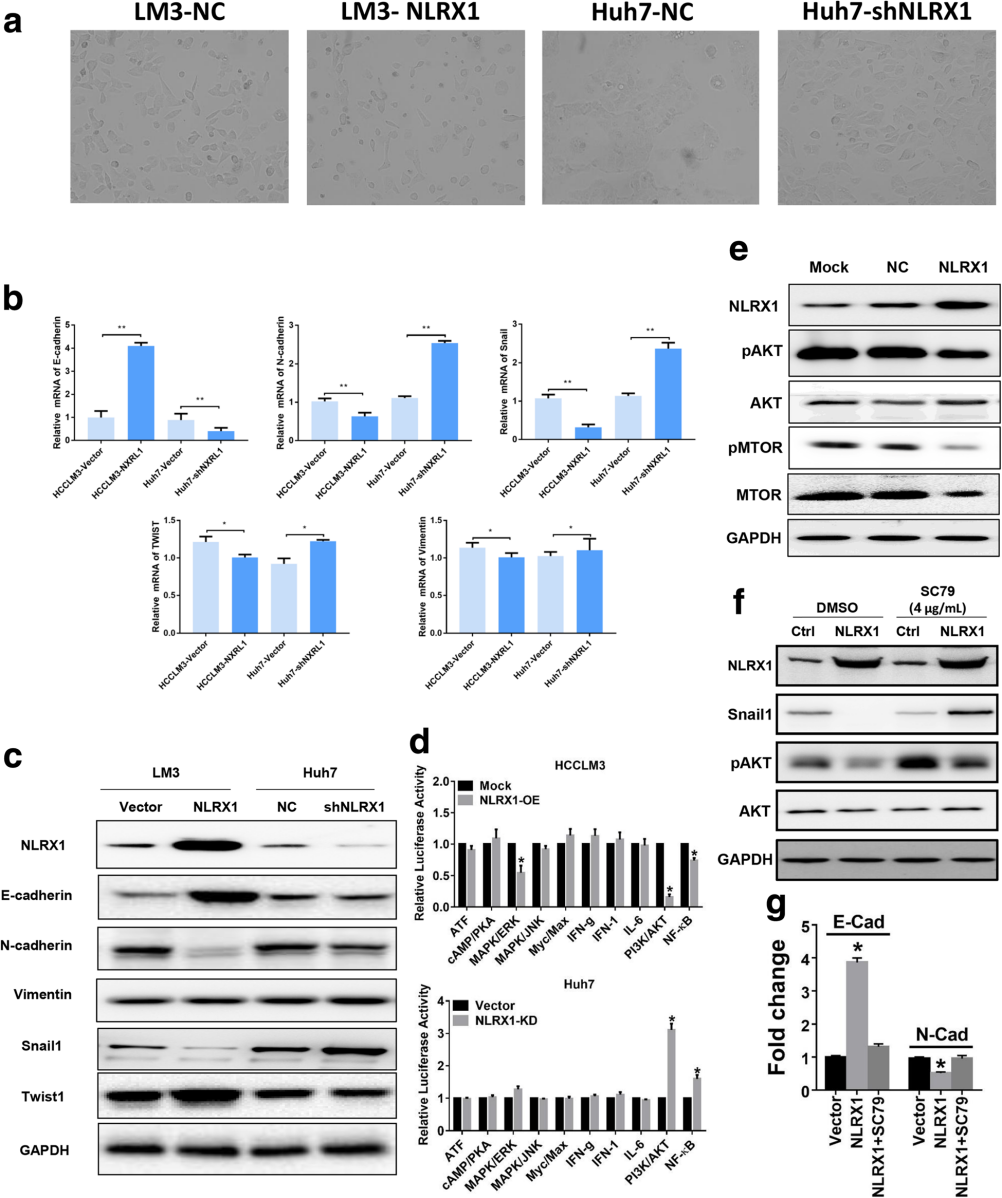
NLRX1 suppresses EMT via the AKT-Snail1 axis. **a** Typical images of HCC cells receiving NLRX1 modulation. **b** Expression of E-cadherin, N-cadherin, Vimentin, Snail1, and Twist1 after NLRX1 modulation in Huh7 and HCCLM3 cells detected by RT-PCR. **c** Expression of E-cadherin, N-cadherin, Vimentin, Snail1, and Twist1 after NLRX1 modulation in Huh7 and HCCLM3 cells detected by WB. **d** Results of Cignal Finder RTK signaling 10-Pathway Reporter array conducted in Huh7 and HCCLM3 cell lines. **e** Phosphorylation of AKT and mTOR after NLRX1 OE in Huh7 cells detected by WB. **f** Evaluation of Snail1 expression level and phosphorylation level of AKT after treating NLRX1-OE HCCLM3 cells with an AKT activator, SC79, by WB assays. **g** Evaluation of E-cadherin and N-cadherin expression levels after treating NLRX1-OE HCCLM3 cells with an AKT activator, SC79, by RT-PCR. Asterisk indicates *P* < 0.05

EMT is a key event that can directly induce tumor invasion and is defined by the loss of epithelial characteristics and acquisition of a mesenchymal phenotype. Hallmarks of this procedure are the loss of E-cadherin expression and upregulation of mesenchymal-related markers such as N-cadherin and vimentin [23, 24]. Therefore, we first observed the cell morphology changes after NLRX1 modulation, and we found that HCCLM3 cells exhibited epithelial-like morphology after NLRX1-OE, while Huh7 cells displayed an elongated spindle shape, distinctive than the rounded appearance in their parental cells after NLRX1 knock-down (Fig. 3a). Next, we performed RT-PCR to evaluate the expression of several EMT-related markers. NLRX1 OE significantly increased mRNA E-cadherin mRNA levels and downregulated N-cadherin, vimentin, Snail1, and Twist1 (Fig. 3b). Meanwhile, NLRX1 KD in Huh7 cells induced an opposite expression pattern (Fig. 3b). WB showed that expression patterns of E-cadherin, N-cadherin, and Snail1 after NLRX1 modification were similar to those observed with RT-PCR. However, Twist1 and vimentin protein levels were not different according to WB (Fig. 3c). These results demonstrated that NLRX1 can successfully suppress EMT in HCC by slightly decreasing Snail1 expression.

To further clarify the mechanism underlying how NLRX1 suppress EMT process, a Cignal Finder RTK signaling 10-Pathway Reporter array was used. Results showed that PI3K-AKT signaling pathway was the most altered pathway due to NLRX1 modulation (Fig. 3d). We further performed a QPCR array including critical genes involved in PI3K-AKT signaling pathway. We found that key genes regulating cell proliferation and migration including P21 (CDNK1A), CCNE2, EIF4E, MDM2, BCL2, and FN1 were significantly upregulated due to NLRX1-KD, while several tumor suppressor genes including TSC2, RPTOR, and TP53 were downregulated due to NLRX-KD in Huh7 cells (Additional file 1: Table S1). Activation of PI3K-AKT signaling is a major event in HCC progression [25–27], and recent studies demonstrated that the PI3K-AKT pathway induces EMT in a Snail-dependent manner [28]; thus, we inferred that PI3K-AKT pathway might be the key downstream pathway of NLRX1 which contributed to the suppression function of NLRX1. Therefore, we further investigated whether NLRX1 could inhibit PI3K-AKT pathway activation. We found that NLRX1-OE HCCLM3 cells exhibited significantly lower levels of phosphorylated AKT compared with control cells (Fig. 3e). Moreover, as a key downstream target of AKT activation, we also examined the activation status of mammalian target or rapamycin (mTOR). NLRX1-OE markedly inhibited the phosphorylation of mTOR (Fig. 3e), indicating that NLRX1 suppresses PI3K-AKT signaling. To validate the critical role of PI3K-AKT signaling pathway, we conducted rescue experiments via using an AKT activator, SC79. We found that re-activating AKT could effectively abolish the inhibition effect of NLRX1-KD on snail1 expression (Fig. 3f). In addition, re-activation of AKT could restore the mesenchymal-like phenotype of HCCLM3 cells regardless of NLRX1 overexpression (Fig. 3g). Taken together, our results suggest that NLRX1 effectively suppresses the EMT process by downregulating Snail1 expression via inhibition of the PI3K-AKT pathway.

### NLRX1 promoted cell senescence by a P21-dependent manner

**Fig. 4:**
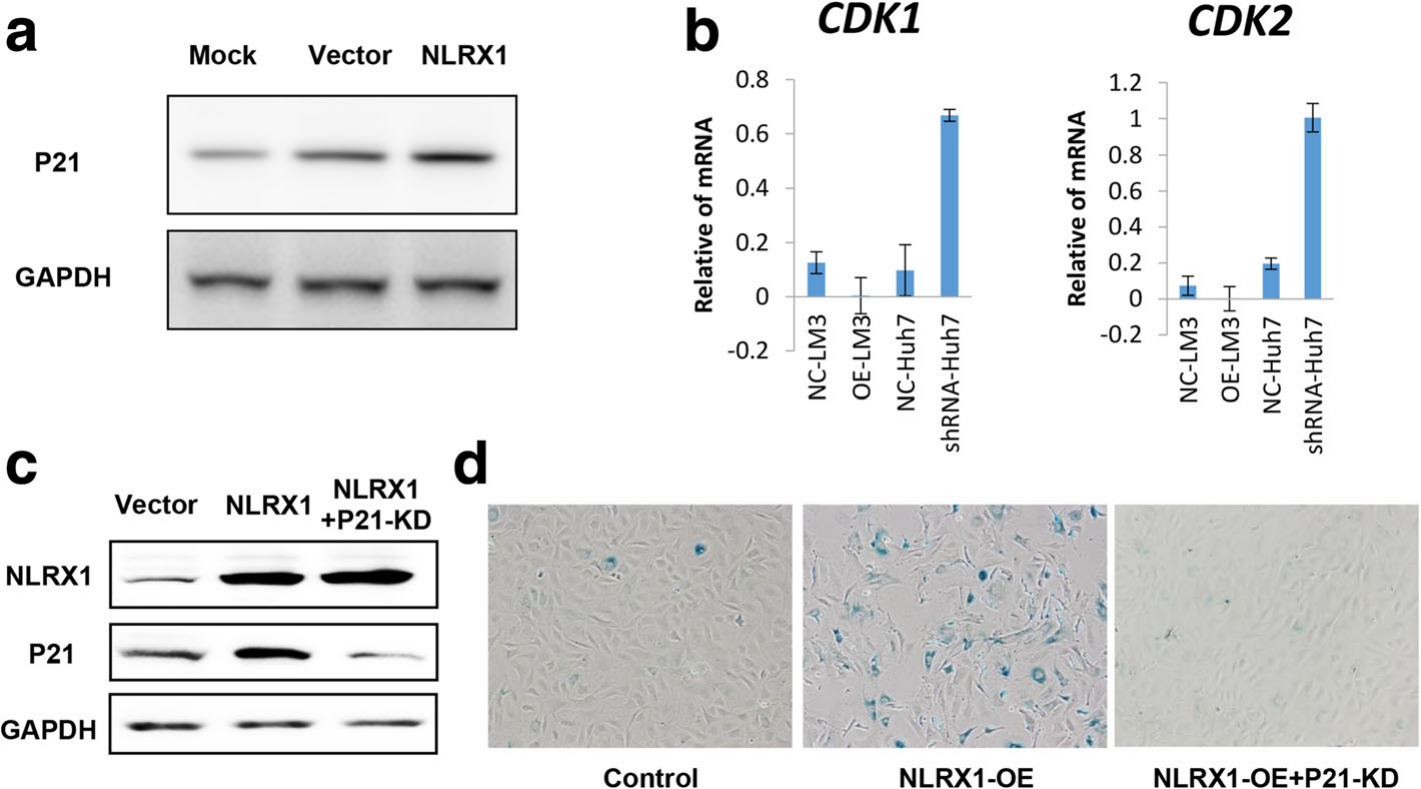
NLRX1 induces cell senescence via a P21-dependent pathway. **a** P21 expression after NLRX1 OE in HCCLM3 cells detected by WB. **b** Evaluation of the expression levels of two downstream targets of P21, CDK1, and CDK2, after NLRX1 modulation. **c** Validation of NLRX1 and P21 modulations in HCCLM3 cells by WB assays. **d** β-galactosidase activity experiments for HCCLM3 cells after different treatments

Cell senescence is considered as a key step in apoptosis, and this pathway is mainly controlled by P21 [29], which is a key downstream target of AKT [30]. Given that NLRX1 can induce HCC cell apoptosis and suppress PI3K-AKT signaling, we further investigated whether NLRX1 promotes cell apoptosis by hindering P21 expression. We found that NLRX1-OE could effectively upregulate P21 expression (Fig. 4a), and expressions of down-stream targets of P21 including CDK1 and CDK2 were significantly repressed due to high level of P21 expression caused by NLRX1 overexpression, while knocking down NLRX1 in Huh7 resulted in increased expression of CDK1 and CDK2 (Fig. 4b). To further validate the role of P21 in NLRX1 induced senescence, we knocked down P21 in NLRX1-OE cells, which was validated by WB assays (Fig. 4c), and performed SA-β-gal activity evaluation. Results showed that NLRX1-OE significantly increased SA-β-gal activity, indicating a higher proportion of aging in NLRX1-OE cells compared to cells transfected with vector, and P21 depletion attenuated the effect of NLRX1 OE (Fig. 4d). Collectively, these data indicate that NLRX1 promotes cell senescence via the PI3K-AKT-P21 axis, thus contributing to HCC cell apoptosis.

### Fragment 556–974 is required for the suppressive function of NLRX1

**Fig. 5:**
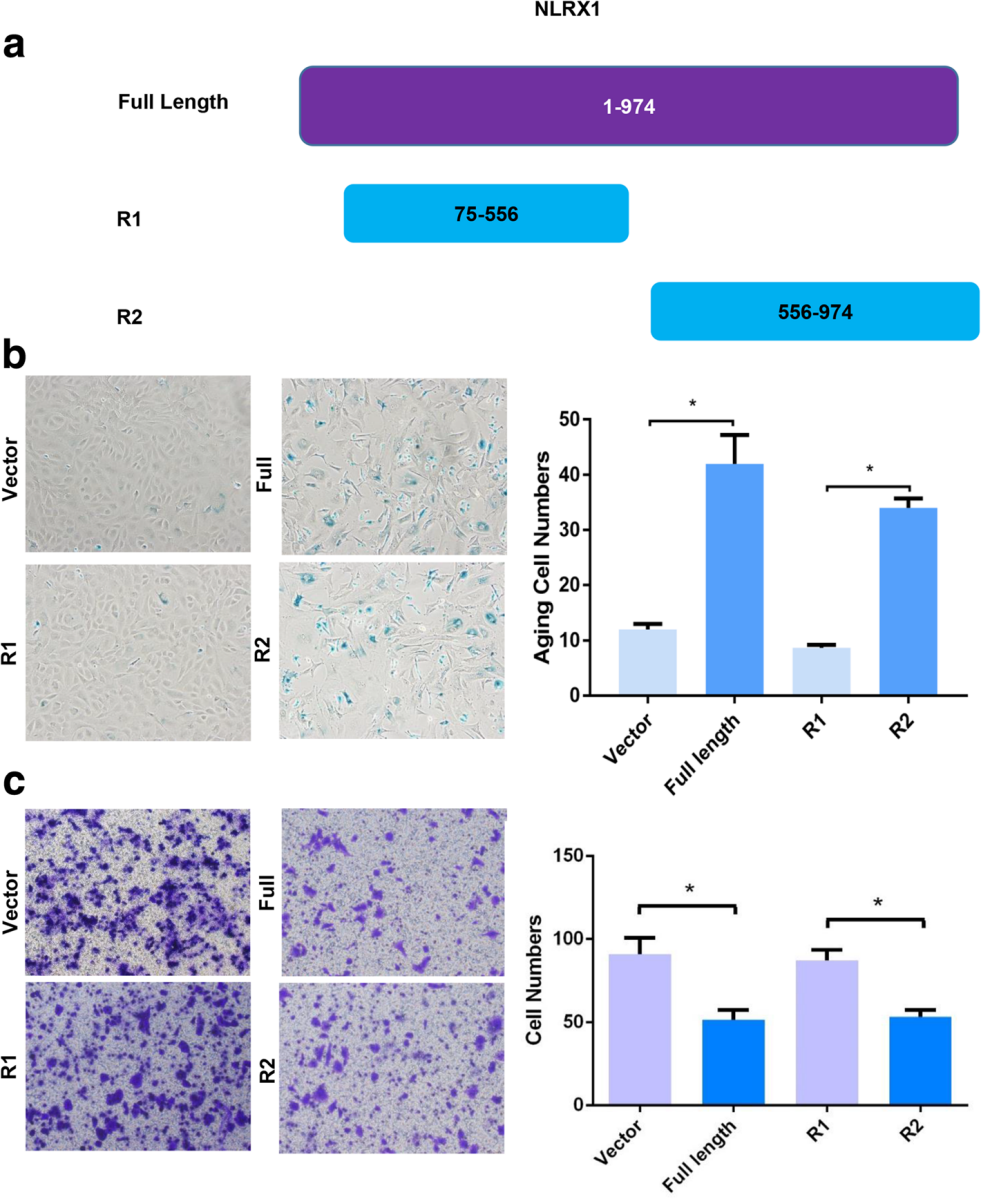
Fragment 556–974 is required for the suppression function of NLRX1. **a** Depictions of full-length and R1 and R2 fragments of NLRX1. **b** β-galactosidase activity experiments for HCCLM3 cells transfected with different NLRX1 fragments. **c** Transwell invasion assays for HCCLM3 cells transfected with different NLRX1 fragments. Asterisk indicates *P* < 0.05

We attempted to identify the critical fragment of NLRX1 that contributes to its suppressive function. We generated three types of plasmids expressing the following peptides: fragment 75–556 (R1), fragment 556–974 (R2), and fragment 1–974 (full length), and transfected them into HCCLM3 cells (Fig. 5a). We found that transfection of the R2 plasmid, but not R1, could induce HCC cell aging similar to expression of the full-length fragment (Fig. 5b). Moreover, Transwell assays showed that R2 plasmid transfection significantly inhibited cell invasion like the full-length fragment; however, the R1 plasmid failed to mimic the effect of full-length NLRX1 (Fig. 5c). Based on these results, the 556–974 fragment of NLRX1 was identified as a critical contributor to its suppressive function.

## Discussion

Previous investigations of NLRX1 mainly focused on the host–pathogen reaction field [16]. However, a growing body of evidence indicates that NLRX1 plays role in regulating metabolism, cell death, and tumorigenesis [14, 20, 21]. Here, we showed that NLRX1 serves as a tumor suppressor in HCC, and its expression is associated with improved prognosis. The present findings support previous studies reporting that NLRX1 attenuates tumor progression. Moreover, our results demonstrate that NLRX1 impairs tumor invasiveness by inhibiting EMT—a critical biological process for HCC progression—and promotes cell senescence in a P21-dependent manner. Mechanistically, PI3K-AKT signaling was identified as the key downstream pathway of NLRX1, and amino acids 556–974 was identified as the key fragment of NLRX1 to execute its suppression function.

Invasiveness is a major characteristic of HCC that contributes to the high incidence rates of recurrence and metastasis, leading to poor patient outcomes [31, 32]. Acquisition of invasion potential is a complex process, and EMT is currently considered as the critical step for this biological transformation [33, 34]. We found that NLRX1 OE induced an epithelial-like phenotype, while NLRX1 KD resulted in a mesenchymal-like phenotype. Moreover, modification of NLRX1 directly affected the invasiveness potential of HCC cells as demonstrated by Transwell assays. These observations show that NLRX1 serve as a key regulator that negatively regulates EMT to prevent tumor progression, and downregulation of NLRX1 reflects a high invasiveness potential and a high tendency towards tumor recurrence or metastasis as observed in the clinical data.

Activation of PI3K-AKT signaling pathway is a hallmark for HCC formation and progression, and reversing this abnormal activation is considered a promising therapeutic approach for HCC [35]. We found that NLRX1 OE greatly repressed AKT phosphorylation, leading to inactivation of downstream kinases and downregulation of target molecules. Importantly, we identified Snail1 as the key downstream target of AKT suppression, revealing a PI3K-AKT-Snail1 axis that is repressed by NLRX1. Our results clarify the mechanism by which NLRX1 negatively controls EMT and suggest a novel, promising target for inhibiting PI3K-AKT signaling to improve the prognosis of patients with HCC. Our next goal is to further investigate the underlying mechanism how NLRX1 modulated PI3K-AKT activation, and this work is ongoing in our lab currently.

Cellular senescence is a biological process that reflects the cellular response to various kinds of stress including dysfunction of survival-related signaling pathways; these cells enter a long-term state of proliferative arrest, which could eventually lead to apoptosis [36, 37]. During this fetal process, the tumor suppressor P21 reportedly plays a vital role in triggering cell cycle arrest, preventing the accumulation of aging cells, which will greatly impair tumor growth [29]. In present study, we observed that NLRX1 OE significantly increased the proportion of aging cells and induced apoptosis. Moreover, tumor growth was greatly impaired by NLRX1 OE during in vivo experiments. We also showed that NLRX1 exerted its pro-apoptotic function through upregulation of P21 following inactivation of PI3K-AKT signaling. Our results provide insight that could lead to novel therapeutic approaches based on re-expressing NLRX1 in patients with HCC.

Our clinical results demonstrate that NLRX1 expression is an independent indicator for both TTR and OS, which suggests that it could be a useful biomarker for the prognosis of patients with HCC. P53 is the main tumor suppressor marker currently measured to predict patient outcome [38]; however, its precision is unsatisfactory. P21 serves as a key downstream effector molecule of P53 that executes its suppressor function, so P21 staining might have greater prediction performance than P53 staining. This comparison is now ongoing in our center.

There are some limitations of our study. First, the detailed interaction between NLRX1 and PI3K-AKT signaling pathway requires deeper investigation. However, our results strongly suggest that NLRX1 inhibits this critical pathway. Second, it will be important to understand why NLRX1 is downregulated, and this work is ongoing in our lab. Moreover, a larger cohort of patients is needed to validate the clinical utility of NLRX1 as a prognostic indicator.

## Conclusions

In summary, our present work identified NLRX1 as a tumor suppressor in HCC. This receptor induces cell apoptosis through promoting senescence and decreases invasiveness by repressing the EMT process. Mechanistically, NLRX1 exerts its suppressive function by inactivating PI3K-AKT signaling. The loss of NLRX1 indicates a poor prognosis in HCC. Future investigations to identify the mechanism(s) underlying NLRX1 loss might provide new insight into HCC treatment.

## Additional file

Additional file 1: **Table S1.** Different expressed genes related to PI3K-AKT signaling pathway in Huh7 cells after NLRX1 knock-down. (DOCX 15 kb)

## Abbreviations

AFP: α-Fetoprotein
BCLC: Barcelona Clinic Liver Cancer
DMEM: Dulbecco’s modified Eagle’s medium
EMT: Epithelial-mesenchymal transition
FBS: Fetal bovine serum
HCC: Hepatocellular carcinoma
IL: Interleukin
KD: Knock-down
NF-κb: Nuclear factor κB
NLRX1: NOD-like receptor X1
NOD: Nucleotide-binding oligomerization domain
OE: Over-expression
OS: Overall survival
PBS: Phosphate-buffered saline
PI3K: Phosphoinositide 3-kinase
RFS: Recurrence-free survival
SDS: Sodium dodecyl sulfate
TMA: Tissue microarray
TTR: Time to recurrence
