# Supplementary material for: NOD-like receptor X1 functions as a tumor suppressor by inhibiting epithelial-mesenchymal transition and inducing aging in hepatocellular carcinoma cells
Source: J Hematol Oncol. 2018 Feb 26;11:28. doi: 10.1186/s13045-018-0573-9 (PMC5828065; doi:10.1186/s13045-018-0573-9)
Supplement: Supplementary file 1 — Table S1. Different expressed genes related to PI3K-AKT signaling pathway in Huh7 cells after NLRX1 knock-down. (DOCX 15 kb) [file 13045_2018_573_MOESM1_ESM.docx]

| **S****upplementary Table 1. Different expressed genes related to PI3K-AKT signaling pathway in Huh7 cells after NLRX1 knock-down** | | |
| --- | --- | --- |
| Symbol | Fold Change | Expressed after NLRX1-KD |
| BCL2 | 6.75 | Up |
| FOXO3 | 3.60 | Up |
| VEGFA | 3.39 | Up |
| MDM2 | 3.35 | Up |
| CCND2 | 3.13 | Up |
| PGF | 3.05 | Up |
| LAMB3 | 2.83 | Up |
| ANGPT1 | 2.68 | Up |
| CREB5 | 2.61 | Up |
| SGK1 | 2.58 | Up |
| MCL1 | 2.58 | Up |
| COL6A3 | 2.41 | Up |
| ITGAV | 2.41 | Up |
| CRTC2 | 2.16 | Up |
| CCNE2 | 2.16 | Up |
| EIF4E | 2.02 | Up |
| LAMA2 | 2.00 | Up |
| TSC1 | 1.94 | Up |
| EPHA2 | 1.89 | Up |
| CCNE1 | 1.89 | Up |
| YWHAE | 1.86 | Up |
| KITLG | 1.77 | Up |
| HSP90AA1 | 1.77 | Up |
| RPS6KB1 | 1.73 | Up |
| RAF1 | 1.70 | Up |
| PIK3R1 | 1.69 | Up |
| AKT2 | 1.68 | Up |
| FN1 | 1.65 | Up |
| PDPK1 | 1.63 | Up |
| MAPK3 | 1.58 | Up |
| TSC2 | -1.50 | Down |
| RBL2 | -1.50 | Down |
| IKBKB | -1.51 | Down |
| PPP2R1B | -1.52 | Down |
| TP53 | -1.52 | Down |
| CDK4 | -1.52 | Down |
| BAD | -1.53 | Down |
| SOS1 | -1.53 | Down |
| GNB5 | -1.53 | Down |
| PKN1 | -1.53 | Down |
| SOS2 | -1.54 | Down |
| OSMR | -1.55 | Down |
| SPP1 | -1.69 | Down |
| PPP2R5D | -1.82 | Down |
| DDIT4 | -1.82 | Down |
| RELN | -1.83 | Down |
| GNG7 | -1.91 | Down |
| IRS1 | -2.03 | Down |
| CDKN1A | -2.09 | Down |
| GYS1 | -2.13 | Down |
| RPTOR | -2.22 | Down |
| GNG4 | -2.28 | Down |
| G6PC | -2.99 | Down |
